# Soil nematodes show a mid-elevation diversity maximum and elevational zonation on Mt. Norikura, Japan

**DOI:** 10.1038/s41598-017-03655-3

**Published:** 2017-06-08

**Authors:** Ke Dong, Itumeleng Moroenyane, Binu Tripathi, Dorsaf Kerfahi, Koichi Takahashi, Naomichi Yamamoto, Choa An, Hyunjun Cho, Jonathan Adams

**Affiliations:** 1 Department of Biological Sciences, College of Natural Sciences, Seoul National University, Seoul, 151-742 South Korea; 2 Institut National de la Recherche Scientifique, Centre INRS-Institut Armand-Frappier, 531 boulevard de Prairies, Laval, Quebec H7V 1B7 Canada; 3 Korean Polar Research Institute, Incheon, Korea; 4 Department of Biology, Faculty of Science, Shinshu University, Matsumoto, Japan; 5 Department of Environmental Health Sciences, Graduate School of Public Health, Seoul National University, Seoul, South Korea

## Abstract

Little is known about how nematode ecology differs across elevational gradients. We investigated the soil nematode community along a ~2,200 m elevational range on Mt. Norikura, Japan, by sequencing the 18S rRNA gene. As with many other groups of organisms, nematode diversity showed a high correlation with elevation, and a maximum in mid-elevations. While elevation itself, in the context of the mid domain effect, could predict the observed unimodal pattern of soil nematode communities along the elevational gradient, mean annual temperature and soil total nitrogen concentration were the best predictors of diversity. We also found nematode community composition showed strong elevational zonation, indicating that a high degree of ecological specialization that may exist in nematodes in relation to elevation-related environmental gradients and certain nematode OTUs had ranges extending across all elevations, and these generalized OTUs made up a greater proportion of the community at high elevations – such that high elevation nematode OTUs had broader elevational ranges on average, providing an example consistent to Rapoport’s elevational hypothesis. This study reveals the potential for using sequencing methods to investigate elevational gradients of small soil organisms, providing a method for rapid investigation of patterns without specialized knowledge in taxonomic identification.

## Introduction

Ecologists have long been fascinated by how communities and individual taxa respond to elevational gradients. Elevational trends in diversity and community structure have been well documented among a wide range of forms of life, including animals, plants, insects or some other larger invertebrates^1–4^. In recent years, elevational trends in community structure and diversity of microorganisms have also been discovered, due to advances in extraction and sequencing of environmental DNA^5–7^. However, there has so far been very little concerted effort to determine how diversity patterns and community structure of soil nematodes vary with elevation, despite their abundance and important roles in ecosystem processes^8^.

Nematodes are considered to be the most abundant animals on Earth^8^. They occupy almost any environment that provides an available source of organic carbon, playing a significant role in nearly all the world’s ecosystems^9^. Because of the key position of nematodes as primary and intermediate consumers in soil food webs, assessing the controls on diversity of nematode community structure can be seen as being of great importance for assessing future environmental change effects^8, 10^.

To date, only one study has focused on diversity patterns and community structure of soil nematodes vary with elevation, finding no evidence of changes in nematode community diversity across a 1,618 m range of elevation in the USA^11^. It is likely that T-RFLP, the low-resolution molecular technique used in this study, greatly limited the applicability of diversity and community structure. Given the lack of existing studies of soil nematode communities in relation to elevational gradients, it is important to add new work on mountain systems to understanding what patterns exist and what environmental factors may cause these patterns. As well as being of interest in the study of nematode ecology, this would be an important contribution to arriving at general patterns and principles in ecology.

Here, we set out to conduct systematic and representative study focusing on soil nematodes, using high resolution molecular techniques over a broad elevational range. Reviewing studies of elevational diversity trends on a broad range of other taxa, Rahbek^12, 13^ found that around half showed a mid-elevation maximum. Since this is the most common elevational pattern, it is most likely that nematode community will also follow the same pattern, showing a diversity maximum at mid-elevations. Aside from the search for underlying mechanisms, we were firstly interested in knowing whether this empirical pattern is still more widespread, occurring in yet another group of organisms. Moreover, we anticipated that the tendency for other groups of organisms to show mid elevation maxima might actually interact with nematode ecology to produce this pattern in nematodes. If the diversity of trophic sources is greater in mid elevations - due to greater taxonomic diversity in a group that is a food source – this can be expected to allow a greater number of nematode niches to coexist. No relevant studies of elevational trends in other groups of soil organisms exist for Norikura, with the exception of archaea which also show a mid-elevation diversity maximum on Norikura^14^. However, on nearby Mt. Fuji, which has a very similar climate and reaches a similar height to Mt. Norikura, there are mid elevation diversity maxima in both bacteria^15^ and fungi^7^, as well as Archaea^6^. As these are groups that many soil nematodes feed on^16^, the greater diversity of these food sources could hypothetically contribute to a mid-elevation maximum in nematode diversity.

A further reason to anticipate a mid-elevation diversity maximum for nematodes is the predominance of a mid-elevation maximum in precipitation across high mountains in central Japan^17^. For example, Mt. Fuji has an annual precipitation maximum at 2,000–2,500 masl^17^. This coincides with the elevation of the predominant cloud layer, and although the elevational trend of precipitation on Norikura has not been measured, the cloud layer likewise tends to occur at around 2,000–2,500 masl (pers. obvns. by the authors). A mid elevation precipitation maximum for Norikura is potentially significant for soil nematodes because they are strongly dependent on moving within the soil water that surrounds and fills spaces between soil particles^18^. Greater precipitation, combined with cooler temperatures in mid elevations, may be expected to give moister – but still aerated - soils that are physiologically favorable to nematodes and their feeding activity. This itself may promote greater diversity of nematodes because there are fewer restrictions of survival and feeding, allowing greater overall population densities even in specialized niches, promoting niche specialization, and thus species diversity. The precipitation/water balance maximum in mid elevations has been suggested as a cause of diversity maxima in other parts of the world, in other groups that are particularly sensitive to water balance^19, 20^.

There has been much discussion of the possible reasons why mid-elevational diversity maxima occur so commonly^21–23^. One explanation for the mid elevation diversity maximum is the ‘community overlap’ hypothesis, which assumes that there are often two relatively distinct environments on a mountain – an ‘upper mountain’ environment subject to more extreme low temperatures or a different precipitation regime (and often above the treeline), and a ‘lower mountain’ environment that has a distinct ecology (and is often by contrast forested)^23^. If each of these two environments has its own distinct set of species, at the transitional zone where they intersect both groups of species will occur together or nearby one another, giving a mid-elevation diversity maximum^23^.

Another widely favoured explanation for mid elevation diversity maxima is the ‘mid-domain effect’ (MDE), which states that if all species ranges are scattered randomly between the limits of the top and bottom of a mountain, there will be a ‘bulge’ of maximum numbers of overlapping species in the mid elevations^21^. A recent advance of MDE theory has been to include a midpoint attractor – a unimodal gradient of environmental favorability, using a Bayesian simulation model to estimate the location and strength of the attractor from empirical species distribution data along the elevations, within geometric constraints^24^. It has been suggested that gradients of environmental favorability, together with the geometric constraints imposed by the base of a mountain and its summit, will more parsimoniously explain elevational species richness patterns.

We were interested in testing whether the diversity pattern of soil nematode communities would differ with elevation and show a mid-elevation maximum, and whether this pattern would be consistent with the leading hypotheses. Besides testing these predictions for overall trends on mountains, we were also interested in a more generalized way in understanding which particular environmental variables contribute to whatever elevational diversity trend exists, since studies have shown that nematode communities are sensitive to the surrounding environment and several environmental variables. For example, salinity, temperature, pH, soil moisture and organic nutrients have been identified as important factors influencing diversity of nematode communities^4, 25^.

We were also interested in investigating the community structure of soil nematode in relation to elevation, testing the hypothesis that niche specialization will produce a succession of communities along elevation gradients. It is known that for many taxa, there are distinct and predictable community assemblages of organisms which occur at particular elevational levels on mountains^26–28^. This is a result of the fact that many types of organism have clearly defined spatial ranges that are linked to climate and the ecosystem conditions that parallel elevation. The overlap of these ranges is what produces the characteristic communities of each elevational zone. Given that this elevational turnover pattern in species and communities is so widespread, we hypothesized that nematodes, too, would have finely adapted environmental niches in relation to particular elevations, and that this would produce a series of distinctive communities along the elevational gradient.

Lastly, considering that empirical range data of OTUs would be collected and used in the simulation of null model in midpoint attractor model, we were interested in testing for the elevational pattern of species ranges in term of Rapoport’s elevational hypothesis. This is a derivative of Rapoport’s rule^29^, which notes that higher latitude species tend to have larger geographical ranges. The elevational variant of this rule predicts that species found at higher elevations will tend to occur over a broader range of elevations than those from lower down the mountain^30^. The pattern has been documented along elevational gradients in a variety of different taxa, and it has been suggested that it occurs because climates at higher elevations are more variable, so species that occur there must be more tolerant of temperature variation - which also translates into larger elevational ranges^30^.

## Results

### Broad taxonomic features of soil nematode community on Mt. Norikura

In total, 55 samples were collected from 11 elevations on the mountain, along elevational isoclines bands separated by ~200 m of elevation. Four samples were excluded from further nematode community analysis owing to failure of amplification of nematode 18S rRNA genes (Supplementary Table 1).

A total of 70,227 high quality 18S rRNA gene sequences were assigned to 1,121 operational taxonomic units (OTUs) at ≥99% similarity level from 51 samples after the removal of low quality, chimeric, and rare sequences. On average, 98.6 OTUs (±4.4 SE) were found in each sample, which were collected along a ~2,200 meters elevational range (Supplementary Tables 2 and 3). The greatest OTU richness was found at 1,105 meters above sea level (masl) with 149 OTUs, while the lowest OTU richness was found at 2,941 masl with 22 OTUs (Supplementary Table 2).

**Figure 1:**
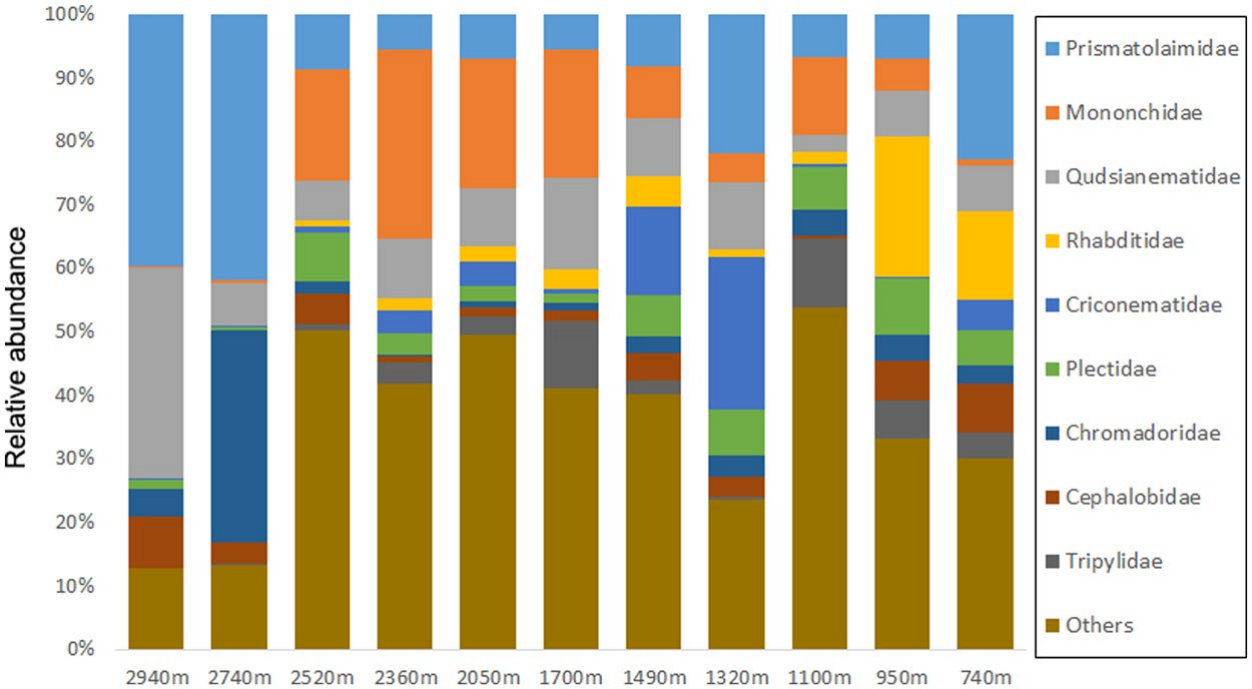
Relative abundance (%) of nematode families on different elevational isoclines, based on total sequence reads.

The nematode community on Mt. Norikura as a whole was dominated by Prismatolaimidae which accounted for 15.3% of the total reads, followed by Mononchidae (10.7%), Qudsianematidae (10.6%), Rhabditidae (5.1%), Criconematidae (5.0%), Plectidae (4.9%), Chromadoridae (4.5%), Cephalobidae (3.9%) and Tripylidae (3.8%). Remaining 18.1% of the total reads were shared by 24 nematode families together, and 17.9% nematode sequences remained unclassified on family level (Fig. 1).

**Figure 2:**
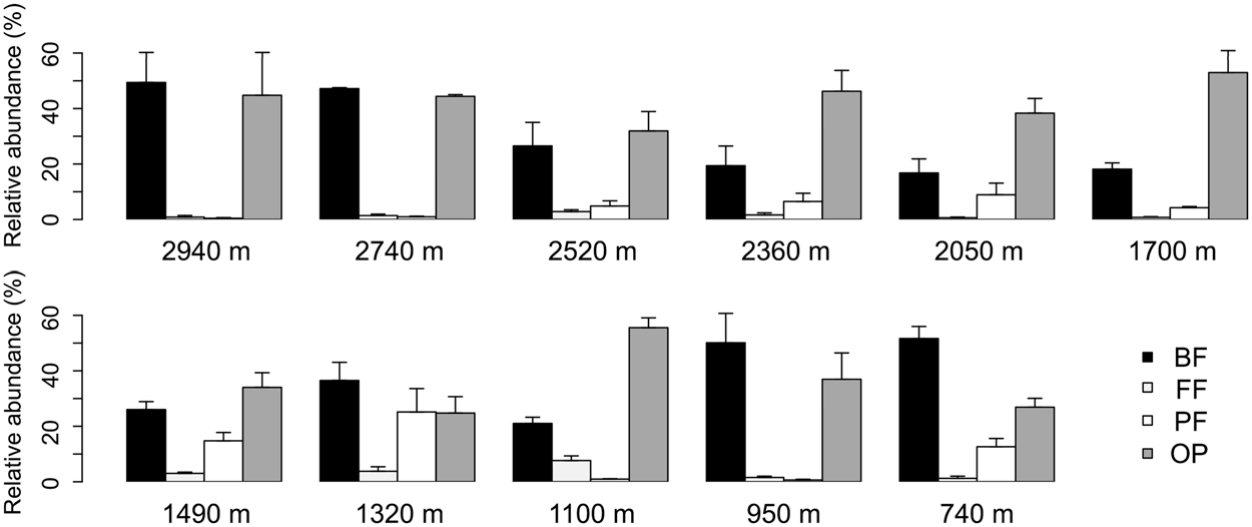
Relative abundance (%) of nematode feeding guilds at eleven different elevations. BF, bacteria feeding; FF, fungi feeding; PF, plant feeding; OP, omnivore/predator. Note the sum of the relative abundance of the four feeding groups is less than 100 percent, due to a sizeable proportion of unclassified sequences.

Classifiable reads were binned into four feeding guilds (bacteria feeding guild [BF], fungi feeding guild [FF], plant feeding guild [PF] and omnivore/predator guild [OP]) according to their family taxonomy and all feeding guilds of nematode were found on Mt. Norikura (Fig. 2). BF and OP together accounted for a major portion of 72.3%, while FF were found low across all elevations of 2.4% in total. The percentage of PF was low on high elevations compared to the lower elevations below 2,500 masl (Fig. 2).

### Elevational change in diversity and community zonation of soil nematodes

**Figure 3:**
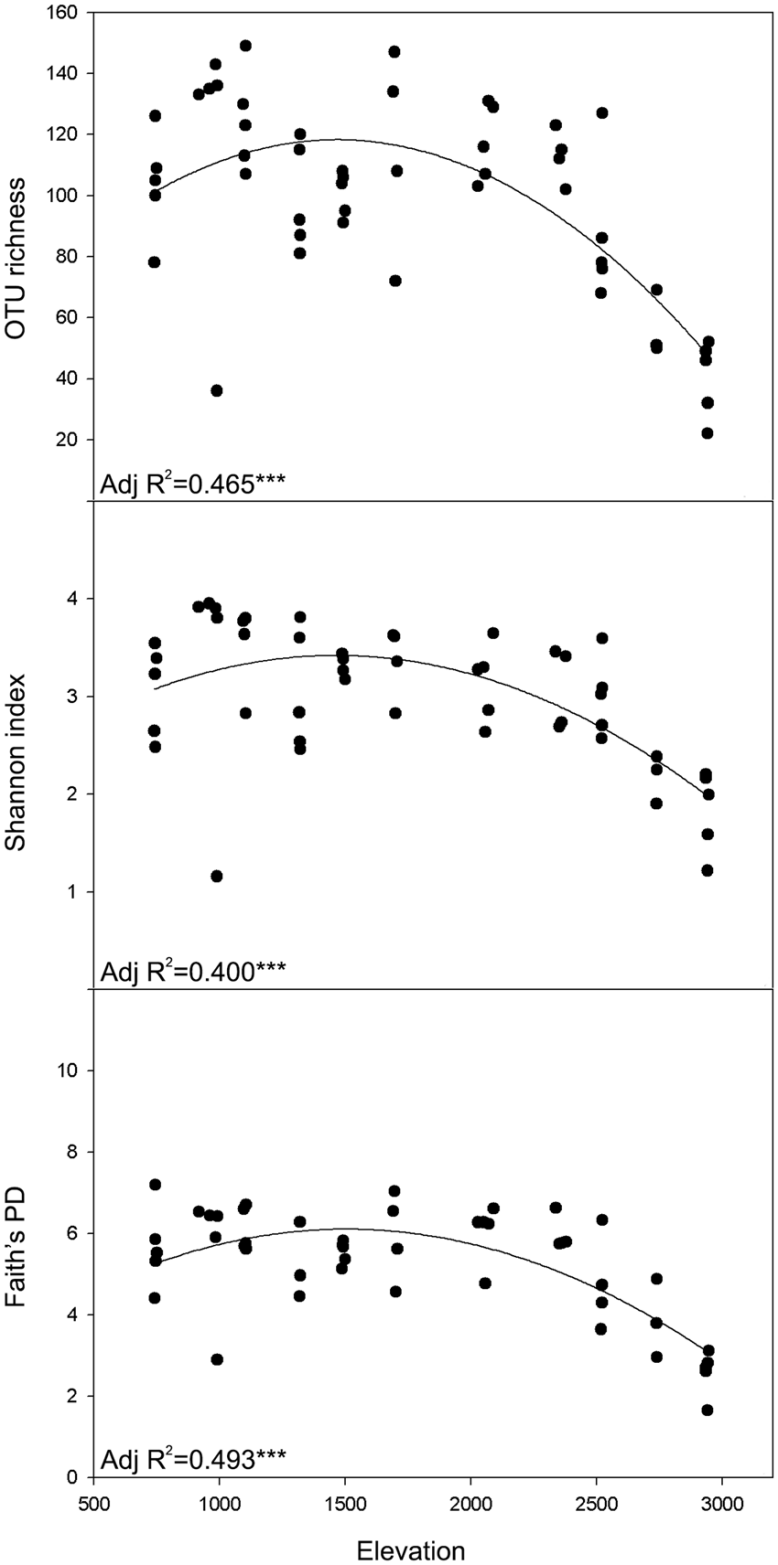
Soil nematode diversity measured along the elevational gradient. OTU richness, Shannon index, Faith’s PD were used. Linear, quadratic and cubic regression models were fitted to assess the relationship of elevation with diversity indices of the nematode communities. Model selection was carried out based on adjusted R^2^ and root mean square error. Only the best fit quadratic regression was shown. Significance levels were less than 0.001, shown as ***. Adj R^2^ is coefficient of determination.

OTU richness, Shannon index and Faith’s PD were used to assess the ecological pattern in nematode diversity. All of the diversity indices were significantly correlated with elevation (p < 0.001), and the richness/diversity showed a mid-elevation maximum, with maximum diversity for all three indices falling between 1,000 and 2,000 masl (Fig. 3). Quadratic models gave the best fit for all three diversity indices (data not shown).

**Figure 4:**
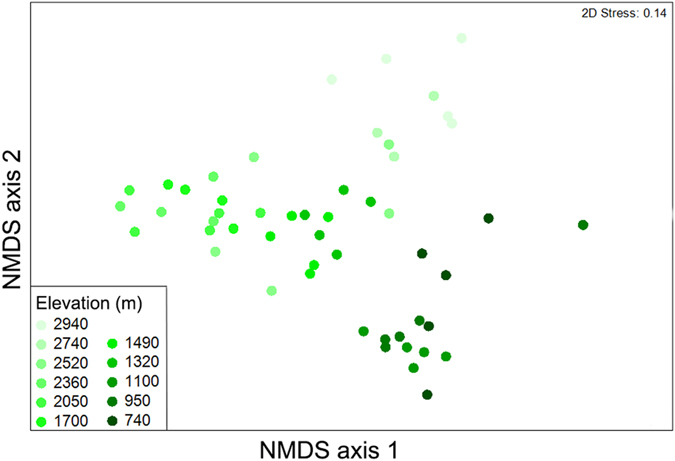
NMDS of Bray Curtis similarity of overall community structure in relation to elevation.

An NMDS performed on the Bray-Curtis similarity matrix of nematode community structure showed significant variability in relation to the elevational gradient (ANOSIM: Global R = 0.761, p < 0.001) (Fig. 4). The samples also formed clear clusters according to the elevation that they came from, visually indicating that samples belonging to different elevational zones harbored distinct communities.

Nestedness analysis indicated that the communities of soil nematodes along the elevational gradient follow a nested structure (p ≪ 0.001). A packed matrix order categorizing nestedness of each sample from low to high was generated, with samples from elevations above 2,500 masl intensively clustered towards the top of the packed matrix order (Supplementary Table 3). This indicates that the communities of higher elevations (above 2,500 m) tend to be a subset of the communities of lower elevations (below 2,500 m).

### Mid domain effect on soil nematode diversity

**Figure 5:**
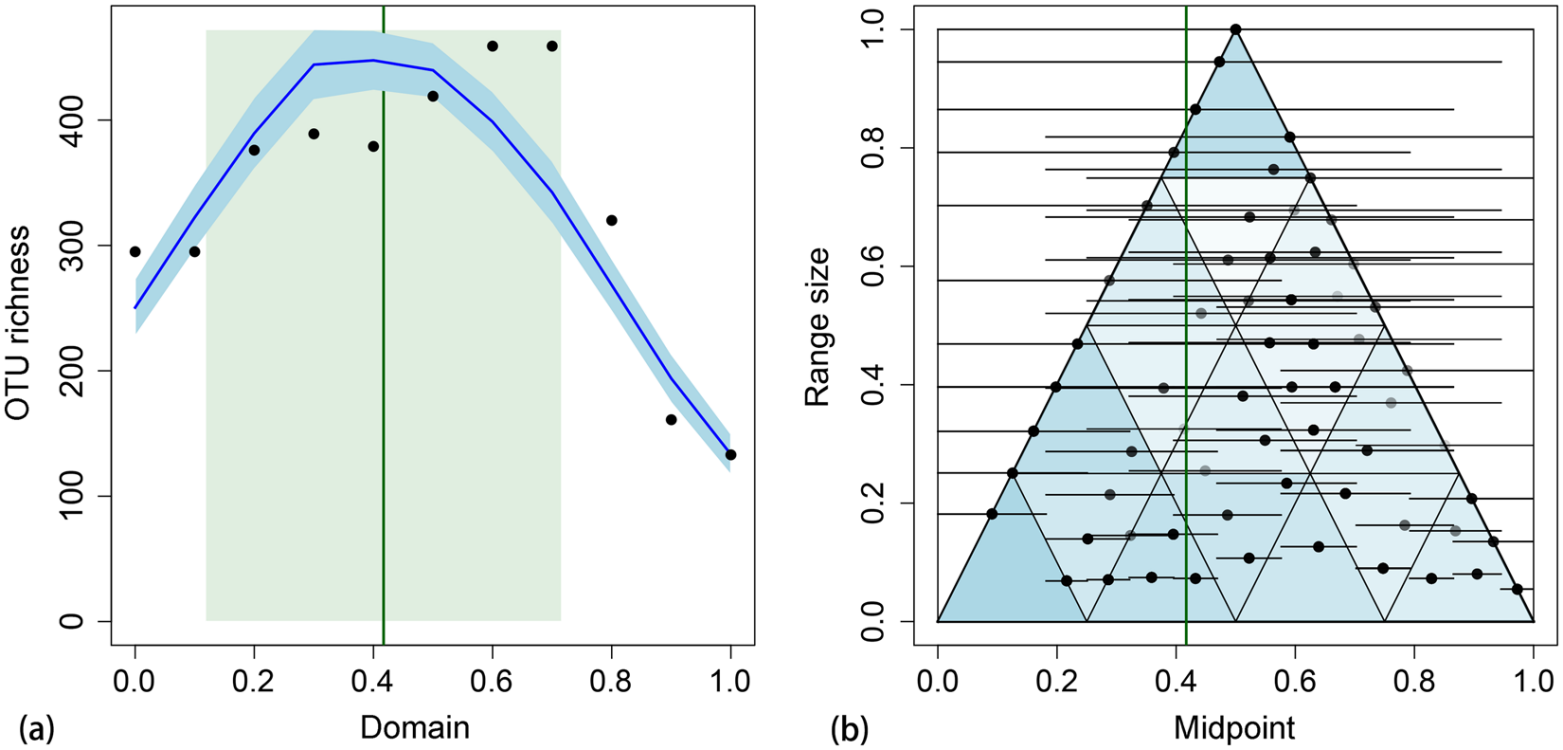
The Bayesian midpoint attractor model applied to the soil nematode data set rescaled to [0, 1] unit line, where 0 represents sea level. (**a**) Mean species richness (dark blue line) and 95% confidence interval (light blue band) for 200 simulations. Dark green vertical line, midpoint attractor; light green rectangle, standard deviation; blue line, modelled OTU richness; black dots, empirical OTU richness. (**b**) Midpoint-range plot for the same data. The x-axis is the location of the range midpoint for each OTU on the elevational domain, and the y-axis plots the elevational span of the range (range size). The large triangle sets the geometrically feasible midpoint limits for ranges of a give size. Black and grey points and associated horizontal line segments illustrate the empirical midpoint and range values for the 1,121 OTUs of soil nematodes. Because many OTUs have identical ranges and midpoints in the data set, the shading of each point is proportional to the number of coincident OTU midpoints. The white-to-blue color scale in the 16 small triangles is proportional to the mean number of modelled points falling in each triangle, averaged over the 200 runs of the simulation.

Each of the empirical ranges of the 1,121 OTUs (mean midpoint of 0.536, mean range of 0.267) was stochastically placed on the domain rescaled from a 3,026 m elevational gradient without replacement of 200 simulations. The result indicated that the simulation is driven by a midpoint attractor at 0.417, with a standard deviation of 0.299 (Fig. 5a). Regression exhibited a good fitness of modeled richness against empirical richness (R^2^ = 0.746, p < 0.001, Supplementary Fig. 1), indicating that gradients in elevational favorability, subject to geometric constraints, may account for the unimodal elevational richness pattern of soil nematodes. Note that the empirical richness, which is underlain by the inferred elevational ranges of OTUs, is not the same concept as the OTU richness of each replicate in Fig. 3. Midpoint and range of OTUs were plotted in Fig. 5b.

### Physicochemical factors influencing soil nematode communities

**Figure 6:**
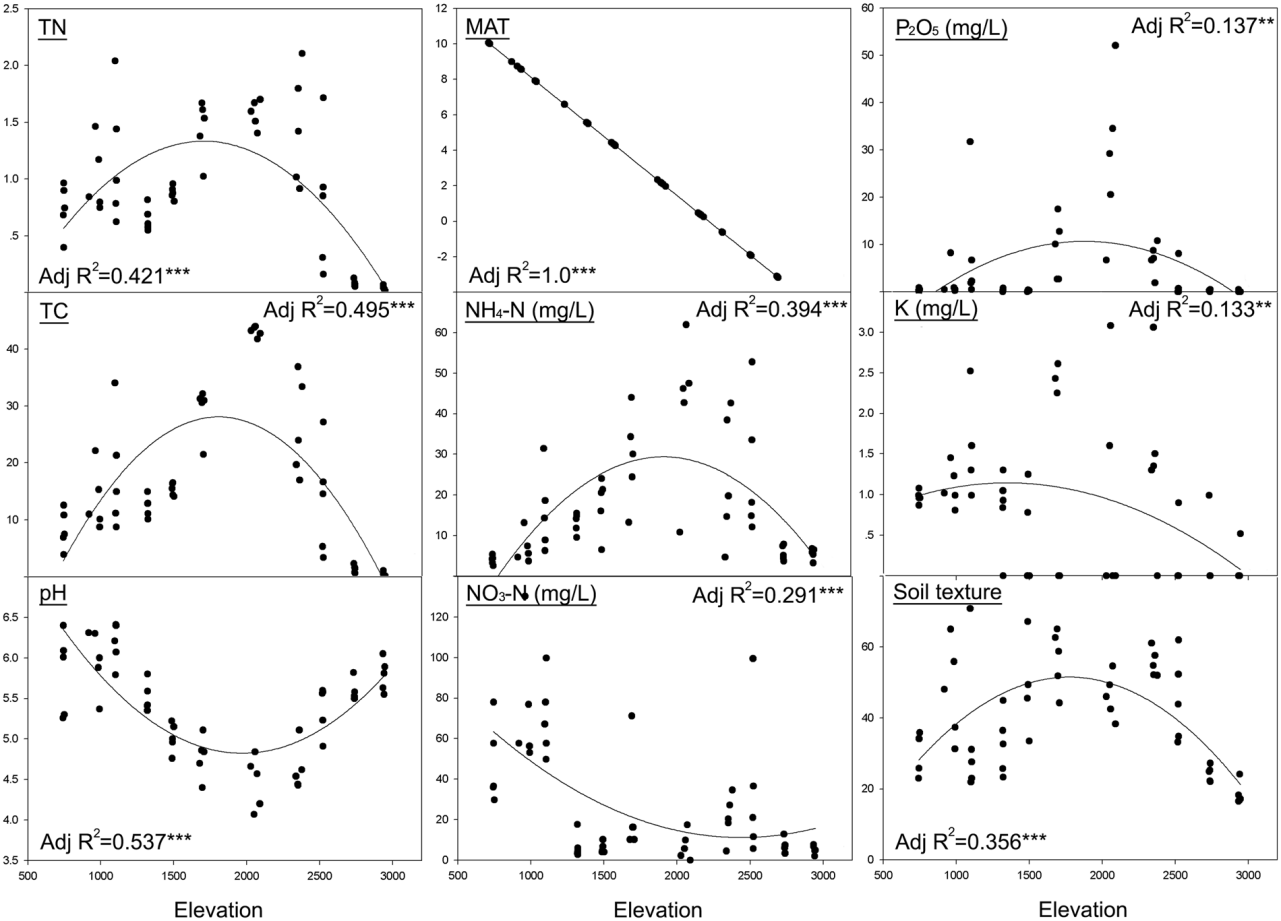
Physicochemical factors shown in relation to elevation. Model selection was carried out based on adjusted R^2^ and root mean square error and only the significant ones are reported. The values of TC and TN are shown in percentages. Total of percentage silt and clay content are used to indicate soil texture.

Nine physicochemical environmental factors for the 55 samples were measured directly, or for elevation calculated on the basis of the moist air lapse rate^31^, with the parameters being statistically different between elevations (p < 0.01) (Supplementary Table 4). Mean annual temperature (MAT), which was calculated using a mean lapse rate of 0.6 °C/100 masl, showed a complete correlation with elevation. The other physicochemical factors including total carbon (TC), total nitrogen (TN), available phosphate (P_2_O_5_), nitrogen in ammonium (NH_4_-N), nitrogen in nitrate (NO_3_-N), potassium (K), pH and soil texture all showed linear unimodal patterns against elevation (Fig. 6).

The multiple regression analysis showed the relative influence of physicochemical factors on nematode diversity indices. Seven variables were tested (see Methods and Materials; Supplementary Fig. 2) and TN and MAT were significantly correlated with all the richness/diversity indices (Table 1).

**Table 1:** Linear model regression between environmental variables and diversity indices.

	OTU richness (R^2^ = 0.614***)	Shannon (R^2^ = 0.432***)	Faith’s PD (R^2^ = 0.602***)
Intercept	57.5***	2.3***	3.57***
MAT	2.8***	0.1**	0.1***
TN	33.6***	0.6***	1.4***

Coefficients are shown for significant predictor variables. Significance level is shown at ***p < 0.001; **p < 0.01 and *p < 0.05. The analysis was performed with all measured variables and only the significant ones are shown. Abbreviations: MAT, mean annual temperature; TN, total nitrogen; TC, total carbon.

**Figure 7:**
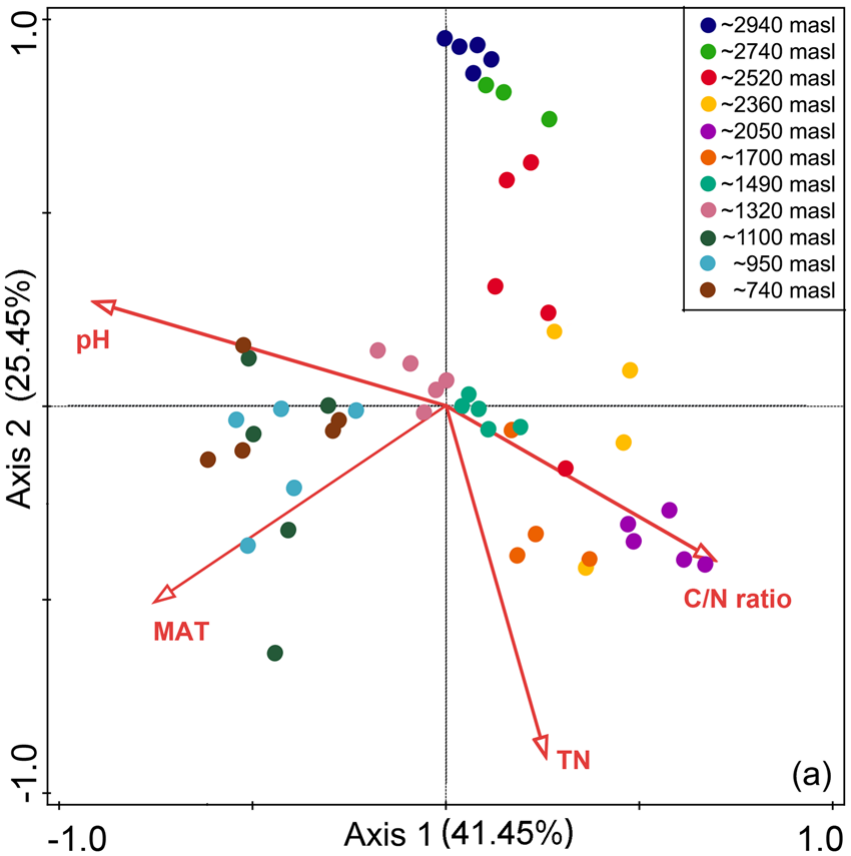
Canonical Correspondence Analysis ordination plot of soil nematode community structure based on 18S gene OTUs and a vector overlay of the environmental variables. Environmental variables were shown in arrows and only the significant ones were presented. Different colors of symbols denote different elevations.

Canonical correspondence analysis (CCA) suggested that among the physicochemical factors relating to elevation, pH (pseudo-F = 3.3, p < 0.005), MAT (pseudo-F = 2.2, p < 0.005), TN (pseudo-F = 1.9, p < 0.005) and C/N ratio (pseudo-F = 1.4, p < 0.005) were the significant contributors to variability of nematode communities on Mt. Norikura (Fig. 7). A total of 66.9% of fitted variation with two axes was explained in an accumulative variance for the interaction between communities and the four significant variables.

### Mean elevational range of nematode OTUs

**Figure 8:**
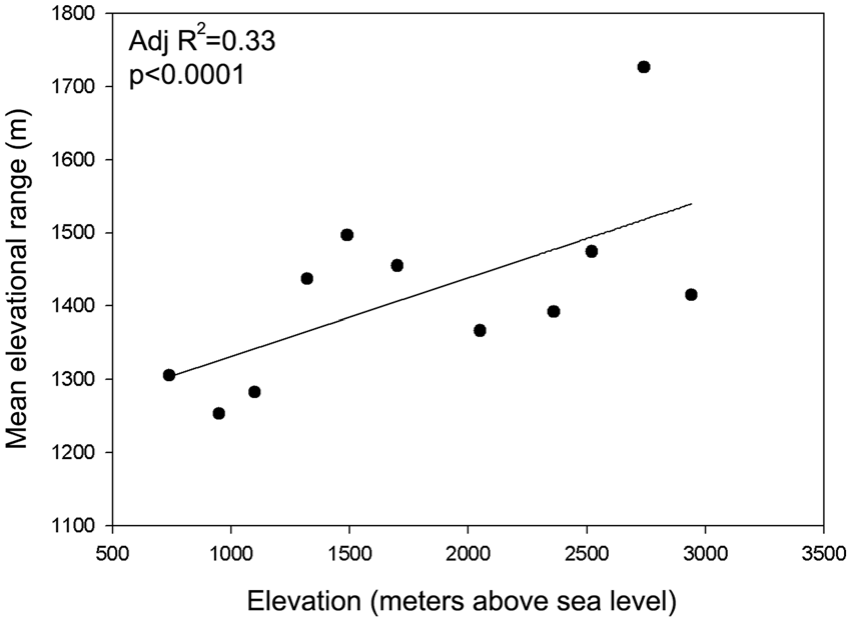
The mean elevational range of high elevation nematodes have broader elevational ranges and therefore broader temperature ranges. High correlations (p < 0.01) were found at all sequence similarities. MAT, mean annual temperature.

Mean elevational range of nematode OTUs was calculated, for each level on the mountain. The average species range extent for nematode community members became greater with increasing elevation (p < 0.01) (Fig. 8).

## Discussion

We found the diversity of soil nematodes was highly correlated with elevation, and showed a mid-elevation maximum on Mt. Norikura. This finding adds a new example to the often-observed unimodal diversity pattern that is found along elevational gradients, although this in itself does not provide an explanation for its occurrence. There are various candidate hypotheses that could explain this pattern.

As explained in the introduction to this paper, mountains in central Japan tend to have rainfall maxima in their mid elevations^17^, and it is possible that the extra soil moisture explains the survival and coexistence of more nematode species in the mid elevations. However, the only available data on precipitation are for Fuji, where the maximum is 2,000–2,500 masl, while maximum diversity of nematodes is only found lower down the mountain at around 1000–2000 masl (Fig. 3). This weakens the case that an elevational gradient in precipitation is a key factor bringing about the observed trend in nematode diversity.

The ‘community overlap’ hypothesis, which assumes that two distinct environments on a mountain each have their own distinct set of species, was obviously not effective in explaining the observed diversity pattern of soil nematode community, because our results indicated that the communities of the lowermost and uppermost elevations on Mt. Norikura are not distinct. Instead, nestedness analysis shows that the communities of higher elevations tend to be a subset of the communities of lower elevations.

On the other hand, the midpoint attractor model, by simulation in a null model, successfully reproduced the empirical richness pattern found on Mt. Norikura, indicating gradients of elevational favorability, subject to geometric constraints, may account for the unimodal elevational richness pattern of soil nematodes. MDE has often been put forward to explain the unimodal patterns found for various taxonomic groups along mountain elevation gradients^6, 22, 32, 33^.

Besides the simulation in null model explaining the ecological diversity pattern, we were also interested in understanding which particular environmental factors may contribute to the deterministic process in terms of ‘niche theory’ and the empirical effects of legacy of past evolution and the current capacity of the system to sustain diversity. Amongst the physicochemical factors we used in analysis of the elevational pattern of nematode diversity, the inferred MAT and measured TN concentration in soil were the strongest predictors of soil nematode diversity, predicting most of the variation. On Mt. Norikura, the TN concentration is greatest in soils in the mid elevations (correlating with the diversity maximum), which – following global scale patterns - may result from slower decay of leaf litter products at moderately cool temperatures^34^, resulting in accumulation of nitrogen in the upper parts of soils, which receive and accumulate partially broken down leaf litter^35^. At lower elevations, with their increased temperatures, more rapid leaf litter decay relative to NPP tends to reduce the N concentrations in the upper soil layers^35^. At higher elevations above 2,500 masl on Norikura, the tree canopy disappears and the vegetation opens up to scrub and then montane tundra^36^. This may also be coupled with drier conditions above the main cloud layer^17^. The lower net primary productivity at the cooler and drier higher elevations, with reduced litter fall more than compensating for slower decay, would explain why TN concentration declines towards upper elevations of Norikura, and with it the diversity of soil nematodes. Although TN was significantly correlated with nematode diversity in this study, greater nitrogen concentration does not always equal greater nematode diversity. Beyond certain levels of surplus nitrogen - for example in farmland ecosystems - nematode diversity decreases^34, 37^. This is thought to be because too much organic nitrogen addition results in very high concentrations of microorganisms, leading to such dense nematode communities that certain nematodes which are adapted of competition between nematode species reduce the overall nematode diversity in that system^34^. Soil temperature (for example MAT) has a marked effect on nematode growth and can significantly influence the composition of nematode communities^38^. Driven by adiabatic cooling, temperature on Mt Norikura is predicted to decline fairly steadily with increasing elevation, starting from the warmest MAT about 10 °C at the lowest elevation site we sampled^39^. While predicted temperature is closely correlated to nematode diversity on Norikura, there is no obvious reason why it should produce a mid-elevation diversity maximum.

We found that the OTU structure of nematode communities was highly variable between different elevations, displaying a clear ‘progression’ of community structure towards successively higher elevations, as is often found for other groups of organisms along elevational gradients^1, 7, 26, 28^. In this study, physicochemical factors of soils and climate were highly correlated with elevation and showed significant influences on community structure of soil nematodes, suggesting that nematodes on Mt. Norikura have quite finely divided niches in relation to the physicochemical factors that vary with elevation. Consistent with previous studies, we found MAT, TN, C/N ratio and pH were all important environmental variables influencing nematode community structure (Fig. 7)^40–42^. This suggests that indeed, nematode OTUs tend to have predictable distributions which are linked to identifiable environmental factors.

When we classified the nematodes on Mt. Norikura in terms of family-level feeding guilds, it was striking that the upper elevations had very few plant feeders, and that the OTUs with very broad elevational ranges belonged to families classified as bacterial feeders or omnivores. This trend could be partly a result of the lack of plant cover at the uppermost elevations on Norikura where an open tundra-like vegetation prevails^36^. While the fungal-based energy channel is one of the main decomposition process in soil^43^, fungal feeders were consistently a minor part of the community over the whole elevational gradient on Mt. Norikura. This may suggest that a faster decomposition model based on the bacterial energy channel predominates on Mt. Norikura.

We calculated the mean elevational ranges of soil nematodes occurring at each 200 m sampling level on the mountain, and found that the mean elevational range for soil nematodes was actually greater in nematode assemblages occurring at higher elevations. The general prediction of a Rapoport pattern as applied to elevational gradients is based on the idea that there will be greater environmental variability on long and short timescales on high mountains^29, 30^. Detailed climatic and microclimatic data for Norikura do not exist, but it is clear that direct heating of the sparsely vegetated upper slopes of mountains can produce very high soil temperatures during the day in summer, followed by drastic cooling at night and during winter^36, 39^. This may require much more generalized physiological niches for soil nematodes. Additionally, if primary productivity decreases with elevation, only species that have very generalized niches may be able to gain enough food to survive at viable population densities at high elevations. The same nematode species may also be able to survive further down the mountain – by virtue of their generalized feeding niches – allowing them to have a wide elevational range. However, the climatic variability hypothesis in explaining elevational range size was rarely based on real climatic data and largely speculative. Though our results were consistent with the Rapoport’s elevational hypothesis, its ecological implications cannot be further interpreted in this limited context of this study.

In conclusion, our study shows that nematodes exhibit various elevational patterns that have been noted from other groups of organisms, indicating how extensive these patterns are in nature. Notably, they may follow a mid elevational diversity maximum, which has been found extensively for many different groups of organisms, and this maximum may follow the deterministic influence of environmental variables as well as the predictions of ‘mid domain effect’ incorporating a mid-point attractor. We also found the nematode OTUs in this study have more extensive elevational ranges prevailing towards higher elevations, which may provide examples to Rapoport’s elevational hypothesis. Further studies are necessary to validate how general these patterns are for nematodes in mountain environments. The modification of the 18s primer for nematodes to use with MiSeq offers a considerable improvement over 454, with much larger yields of sequence data.

## Methods

### Description of sampling sites

Our study site was Mt. Norikura, an extinct volcano in central Japan. Mt. Norikura reaches 3,026 masl and has a cool temperate monsoon climate at its base (MAT of 8.5 °C at 1,000 masl) with abundant year-round precipitation (2,206 mm at 1,000 masl) concentrated in summer^36, 39^. Its surface covering of soils is uniformly derived from andesitic ash deposited over underlying lavas in a series of large eruptions around 15,000 years ago^36, 39^. Mt. Norikura has since then undergone natural ecological succession to give a series of vegetation zones: a montane deciduous broad-leaved forest zone between 800 and 1,600 masl; a subalpine coniferous forest zone between 1,600 and 2,500 masl; an alpine dwarf pine *Pinus pumila* scrub zone between 2,500 and 2,700 masl: and an open herbaceous cushion plant tundra up to about 3,000 m^36^.

### Sampling and gathering soil nematodes

Sampling was carried out over ten days from late July to early August 2014. In total, 55 samples were collected from 11 elevations on the mountain, along elevational isoclines separated by ~200 m of elevation. At each elevational level, five separate samples spaced 100 m apart in a line were taken. Within each individual sample, five soil cores were combined to make a composite sample. The soil cores were 10 cm in diameter and 10 cm deep and were taken at four corners and central point of the quadrat of each sample (Supplementary Fig. 3). Samples were transported to the laboratory at Shinsu University within 5 hours of being gathered. At the laboratory, the contents of each bag were gently mixed, then gently passed through a 5mm sieve.

Nematodes were collected using a modified Baermann funnel method, with nematodes travelling downwards through waterlogged soil and being collected in a tube below^44^. Immediately on arrival of the soil sample at the laboratory, 200 g of soil was wrapped loosely with 2 mm medical gauze and placed into a funnel, which was then filled with distilled water at 20 °C, with the apparatus kept in an air conditioned room at 20 °C during extraction. After 24 hours, 30 ml water in the bottom of the funnel, containing both nematodes and soil particles was collected to centrifuge and the sediment was used to extract nematode DNA.

### Soil analyses

Soil analysis of each sample was carried out at Shinsu University, using standard SSSA protocols (Soil Science Society of America). The parameters analyzed were TC, TN, P_2_O_5_, NH_4_-N, NO_3_-N, K, pH, soil texture, MAT and C/N ratio. Total percentage of silt and clay content are used to indicate soil texture in this paper. MAT was calculated by using a mean lapse rate of 0.6 °C /100 masl, so it showed a complete correlation with elevation.

### DNA extraction, PCR amplification and sequencing of 18S rRNA gene

DNA was extracted using the MoBio Power Soil DNA isolation kit (MoBio Laboratories, Carlsbad, CA, USA) following manufacturer’s instructions, and random empty vials were chosen and run to serve as controls. The isolated DNA was stored at −80 °C until the PCR stage.

Miseq sequencing was used to assess the nematode community through massively parallel sequencing of the 18S rRNA gene. A 350-bp region of the 18S small-subunit rRNA gene was amplified with the primer pairs NF1 and 18Sr2b^45^ with adapter sequences for the Illumina MiSeq. Polymerase chain reaction was performed in a 50-μl reaction mixture composed of 1 or 2 μl of DNA extract, 0.4 μM of each primer, 0.2 mM of each dNTP mix, 1X Taq Reaction Buffer and 1.25 U Solg^TM^ Taq DNA Polymerase (SolGent co., Ltd., Korea). PCR conditions were 10 min at 95 °C, followed by 30 cycles of 60 s at 95 °C, 45 s at 50 °C, and 180 s at 72 °C. Final elongation was at 72 °C for 10 min using the C1000^TM^ thermal cycler (Bio-Rad Laboratories, Inc., Hercules, CA, USA).

Index PCR was performed for the purified PCR products using the Nextera XT Index kit (Illumina, Inc., San Diego, CA, USA). Each of the 50-μl reaction mixtures was composed of 5 μl of each index primer, 2 × PCR Solution Premix Taq^TM^ DNA polymerase (Takara Bio Inc., Otsu, Shiga, Japan), and 5 μl of the purified DNA. PCR conditions were 3 min at 95 °C, followed by 10 cycles of 30 s at 95 °C, 30 s at 55 °C, and 30 s at 72 °C. Final elongation was at 72 °C for 5 min.

After purification by the AMPure XP beads (Beckman Coulter, Inc., Brea, CA, USA), each amplicon was normalized to 4 nM with 10 mM Tris–HCl (pH = 8.5) and pooled with an internal control PhiX (30%). Heat-denatured pooled amplicons were loaded to a v3 600 cycle-kit reagent cartridge (Illumina, Inc.), and 2 × 300 bp paired-end sequencing was performed by the Illumina MiSeq at the Graduate School of Public Health, Seoul National University.

### DNA analysis

The sequences were demultiplexed and trimmed with a read quality score above 20 by the MiSeq Reporter v2.5 (Illumina, Inc.). The sequence data obtained was processed following the Miseq SOP in Mothur^46^. Sequences with any ambiguous bases, sequences with more than 8 homopolymers and sequences with lengths less than 200 bp were removed using the screen. Seqs command in Mothur. Putative chimeric sequences were detected and removed via the Chimera Uchime algorithm contained within Mothur in de novo mode. Rare sequences (less than 10 reads) were removed to avoid the risk of including spurious reads generated by sequencing errors^47^.High quality sequences were assigned to OTUs at ≥99% similarity level.

Taxonomic classification of each OTU was obtained by classifying alignments against SILVA Release 115 databases^48^ using the classify command in Mothur at 80% cutoff with 1000 iterations. The Miseq sequence data used in this study are deposited in the MG-RAST server (Meyer *et al*., 2008) under project ID 17384 (http://metagenomics.anl.gov/linkin.cgi?project=17384).

### Mid domain effect on soil nematode diversity

Colwell’s midpoint attractor model^31^, which simulates the interaction between a unimodal gradient of elevational favorability and the geometric constraints imposed by domain limits, was applied to explain the MDE on the elevational diversity gradient of soil nematode communities. The midpoint attractor model uses two parameters A ([0 < A < 1], the mean location on the unit line domain) and B ([0 < B < 1], the standard deviation around the location) to describe the elevational favorability (attractor).

To model the expected pattern of species richness under the influence of the attractor, each of the empirical ranges in a data set is placed on the domain stochastically, without replacement of 200 simulations, with its midpoint drawn at random from a proposed attractor distribution N (A, B). Algorithm 2^31^ were used for placing ranges within the domain. To enforce the geometric constraint and maintain the empirical range size frequency distribution, the midpoint is sampled from this distribution only over the interval of feasible midpoints, given the size of each range, such that the range does not extend beyond either the lower or upper domain limit (Colwell, 2000; Supplementary Appendix).

### Statistical analysis

We used ANOVA for normal data and Kruskall-Wallis tests for non-normal data to test whether physicochemical factors differed among different elevations. As the midpoint attractor model does not incorporate physicochemical factors into the estimation of the parameters, to evaluate in more detail the effect of the seven remaining physicochemical factors on OTU richness, Shannon Index and Faith’s PD, we performed multiple regression analyses. Non-significant predictor variables were removed sequentially until only significant variables were left in the model. Before applying multiple regression to the dataset, we looked for redundant physicochemical factors using the Varclus procedure in the Hmisc package^49^ in the R platform. High correlation was found between TN and TC (Spearman’s *ρ* ^2^ ≥ 0.91), between TN and P_2_O_5_ (Spearman’s *ρ* ^2^ ≥ 0.73) and between TN and soil texture (Spearman’s *ρ* ^2^ ≥ 0.61; Supplementary Fig. 2). Therefore, we removed TC, P_2_O_5_ and soil texture from the analysis, and used the remaining seven physicochemical factors, {i.e. TN, NH_4_-N, NO_3_-N, K, pH, MAT and C/N ratio} for multiple regression analyses.

Diversity indices such as Shannon Index and Faith’s PD and OTU richness were calculated using the Mothur platform^46^. To assess the best fitting model of correlations between elevation and richness/diversity and environmental variables, linear and polynomial (quadratic) models were tried out using SigmaPlot v 10.0 (Systat Software, San Jose, CA). Model selection was carried out based on adjusted R^2^ and RMSE (root mean square error).

We performed a Non-metric Multidimensional Scaling (NMDS) using Primer v6 to explore patterns in community structure using a Bray Curtis similarity matrix. Abundance data of OTUs were square root transformed to calculate the distance between soil nematode communities. Community structure was compared between elevations using ANOSIM with 999 random permutations.

To individually assess the influence of physicochemical factors on community structure of soil nematode, CCA was used in CANOCO ver. 5. Forward selection was used to select significant explanatory variables with 999 permutations and only significant (p < 0.05) variables were included in the models.

Nestedness analysis was performed by BINMATNEST with default input parameters^50^ to test whether the community of one set samples tending to be a subset of the community present in another set of samples. The significance value of nestedness was tested using default input parameters and null model 3 which calculates the p-value for row and column totals following Dong *et al*.^51^. The samples were re-ordered following the packed matrix order which indicates a high-to-low categorized nestedness.

Furthermore, we investigated the relative abundance of the dominant nematode families across all sites. From their sequence reads, nematodes were identified to family level and binned into their respective functional feeding guilds using previous published comprehensive lists^16^. Four feeding groups were created: bacteria feeding, fungi feeding, plant feeding, and omnivore/predator.

## Electronic supplementary material

supplementary information

supplementary data
