# Supplementary material for: Soil nematodes show a mid-elevation diversity maximum and elevational zonation on Mt. Norikura, Japan
Source: Sci Rep. 2017 Jun 8;7:3028. doi: 10.1038/s41598-017-03655-3 (PMC5465078; doi:10.1038/s41598-017-03655-3)
Supplement: Supplementary file 1 — supplementary information [file 41598_2017_3655_MOESM1_ESM.pdf]

Soil nematodes show a mid-elevation diversity maximum and elevational zonation on Mt. Norikura, Japan

Ke Dong[1,5], Itumeleng Moroenyane[1,2], Binu Tripathi[1,3], Dorsaf Kerfahi[1], Koichi Takahashi[4], Naomichi Yamamoto[5], Choa An[5], Hyunjun Cho[1], Jonathan Adams[1,*]

1, Department of Biological Sciences, College of Natural Sciences, Seoul National University, Seoul 151-742, South Korea

2, Institut National de la Recherche Scientifique, Centre INRS-Institut Armand-Frappier, 531 boulevard de Prairies, Laval, Quebec, H7V 1B7, Canada

3, Korean Polar Research Institute, Incheon, Korea

4, Department of Biology, Faculty of Science, Shinshu University, Matsumoto, Japan

5, Department of Environmental Health Sciences, Graduate School of Public Health, Seoul National University, Seoul, South Korea

*Corresponding author: Jonathan Adams, Department of Biological Sciences, College of Natural Sciences, Seoul National University, Seoul 151-742, South Korea, (TEL) 82-2-880-4339, (Fax) 82-2-880-4962, (Email) geograph.ecol@gmail.com

Key words: elevational trend, nematode community, high-throughput sequencing, Rapoport’s elevational rule

Running title: Elevational trends in soil nematodes

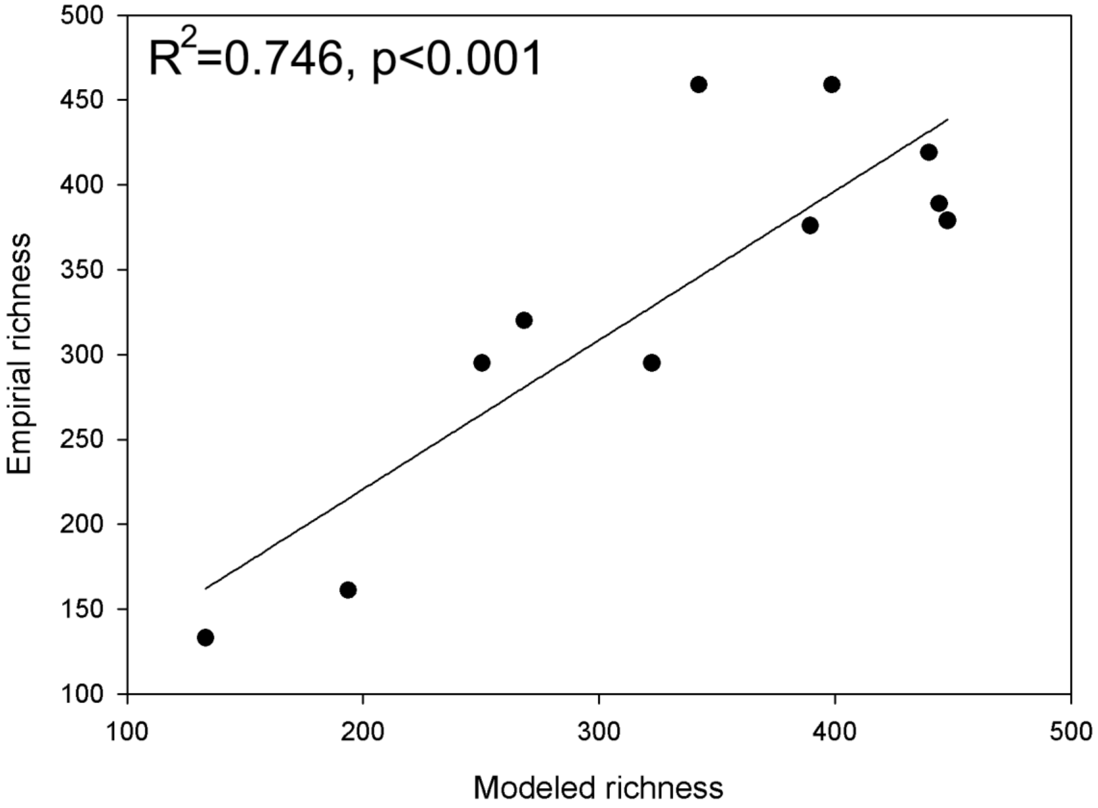

Supplementary Fig.1 Relationship between the simulated richness of soil nematodes and empirical richness based on OTU ranges.

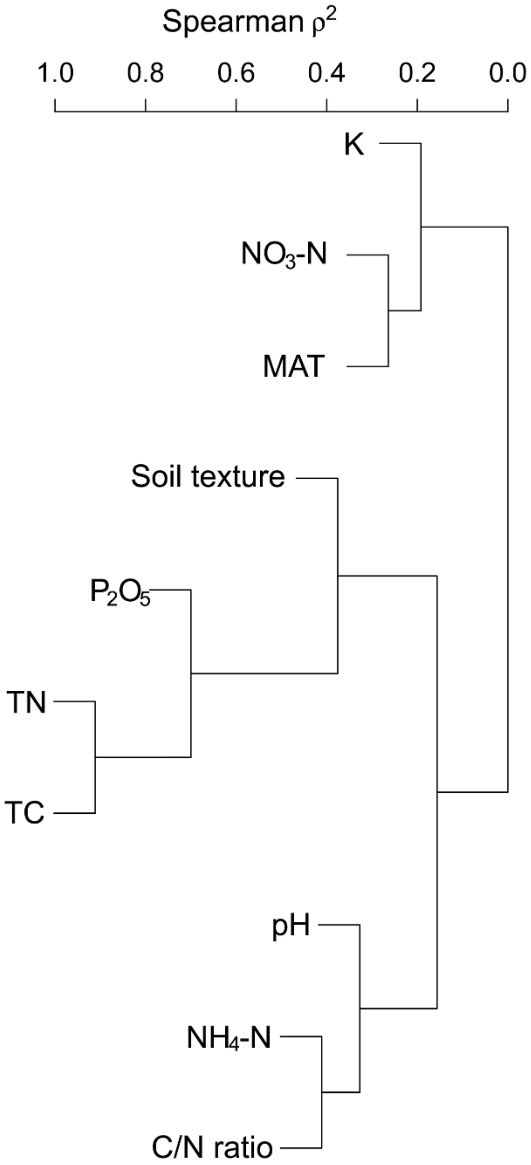

Supplementary Fig.2 Cluster analysis of all measured environmental variables. Covariables with high correlation (TN and TC [Spearman's $\rho^2 \geq 0.91$], TN and P_2O_5 [Spearman's $\rho^2 \geq 0.73$] and TN and soil texture [Spearman's $\rho^2 \geq 0.61$]) were removed on the basis of VARCLUS results.

Abbreviations: NH_4-N, nitrogen in ammonium; C/N ratio: Carbon/Nitrogen ratio; P_2O_5, available phosphate, TN: Total Nitrogen; TC, Total Carbon; NO_3-N, nitrogen in nitrate; K, potassium concentration. Total of percentage silt and clay content are used to indicate soil texture.

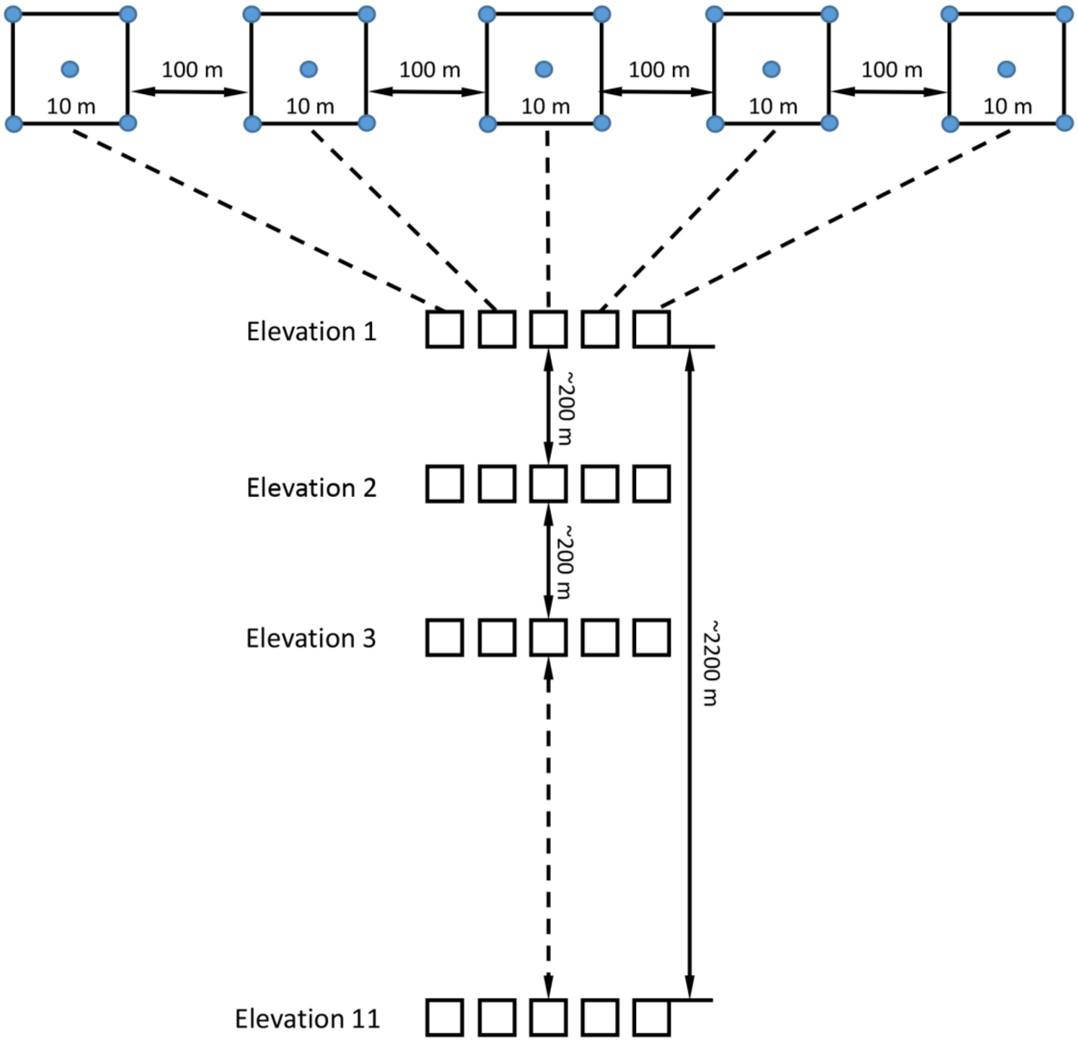

Supplementary Fig. 3 Sampling scheme. Samples were collected from 11 elevations of the mountain, separated by about 200 m elevation. At each elevational level, five quadrats (10 x 10 m in size) were collected 100 m apart along a linear transect. Soil collected from the four corners and center of the each quadrat were pooled to make one sample for DNA extraction and soil property analysis.

Appendix: Defining elevational ranges of OTUs

We calculated the elevational ranges of OTUs following Colwell (2016). If the highest elevation at which an OTU was recorded was not at the highest sampling location, the upper boundary for that OTU range was estimated to occur halfway between the highest elevation of recorded occurrence and the next higher sampling elevation. If the highest elevation at which an OTU was recorded at was the highest sampling elevation, the upper boundary of that species range was estimated to occur halfway between that sampling elevation and the upper limit of the domain. The lower boundary for each range was treated analogously, being extended halfway to the next lower sampling elevation or halfway to the lower domain limit (sea level), if an OTU was recorded at the lowest sampling elevation, but that sampling elevation was not the domain limit. The ranges of each OTU found at only one sampling elevation were treated similarly; otherwise, these point ranges would have had a zero range, and would have been lost from the model. We assumed that the occurrence of each species was continuous between its estimated upper and lower recorded range boundaries.
